# Rank-In Integrated Machine Learning and Bioinformatic Analysis Identified the Key Genes in HFPO-DA (GenX) Exposure to Human, Mouse, and Rat Organisms

**DOI:** 10.3390/toxics12070516

**Published:** 2024-07-18

**Authors:** Xinyang Li, Hua Xiao, Liye Zhu, Qisijing Liu, Bowei Zhang, Jin Wang, Jing Wu, Yaxiong Song, Shuo Wang

**Affiliations:** Tianjin Key Laboratory of Food Science and Health, Research Institute of Public Health, School of Medicine, Nankai University, No.94 Weijin Road, Tianjin 300071, China; lixinyang@nankai.edu.cn (X.L.); 18770620136@163.com (H.X.); zhuliye@nankai.edu.cn (L.Z.); liuqisijing@126.com (Q.L.); bwzhang@nankai.edu.cn (B.Z.); wangjin@nankai.edu.cn (J.W.); wujing2020@nankai.edu.cn (J.W.); ysong90@nankai.edu.cn (Y.S.)

**Keywords:** HFPO-DA, GenX, bioinformatics, transcriptome analysis, machine learning

## Abstract

Hexafluoropropylene Oxide Dimer Acid (HFPO-DA or GenX) is a pervasive perfluorinated compound with scant understood toxic effects. Toxicological studies on GenX have been conducted using animal models. To research deeper into the potential toxicity of GenX in humans and animals, we undertook a comprehensive analysis of transcriptome datasets across different species. A rank-in approach was utilized to merge different transcriptome datasets, and machine learning algorithms were employed to identify key genetic mechanisms common among various species and humans. We identified seven genes—TTR, ATP6V1B1, EPHX1, ITIH3, ATXN10, UBXN1, and HPX—as potential variables for classification of GenX-exposed samples, and the seven genes were verified in separate datasets of human, mouse, and rat samples. Bioinformatic analysis of the gene dataset further revealed that mitochondrial function and metabolic processes may be modulated by GenX through these key genes. Our findings provide insights into the underlying genetic mechanisms and toxicological impacts of GenX exposure across different species and offer valuable references for future studies using animal models to examine human exposure to GenX.

## 1. Introduction

Hexafluoropropylene Oxide Dimer Acid (HFPO-DA, also known as GenX) is among the class of perfluorinated compounds that have been used as replacements for perfluorooctanoic acid (PFOA) and related chemicals in industrial processes [1]. These compounds are characterized by their strong resistance to heat, oil, and water, making them ideal for use in various applications such as firefighting foams, surface coatings, and lubricants [2]. Concerns have arisen regarding the environmental persistence and potential toxicity of GenX compounds, with their growing and wide usage [3]. Studies have indicated that GenX can accumulate in the environment and in the bodies of animals and humans, leading to health risks such as developmental effects, liver toxicity, and metabolic disorders [4,5,6]. Therefore, the exploration of the health risks of GenX is rapidly emerging as a hotspot in toxicological research. However, given that GenX is a substance with low toxicity and a widespread distribution, research on the sensitivity to toxic effects and key biomolecular mechanisms is still restricted and remains unclear. At the same time, toxicological research on GenX is often carried out in animal experiments, but there is still a gap between the findings in animal experiments and their extrapolation to human organisms. Therefore, there is an urgent need to develop rapid and efficient methods to study the toxic effects and sensitive endpoints of GenX toxicity and to extrapolate from animals to humans.

In the field of toxicological research, bioinformatics and toxicogenomics have emerged as crucial research tools for investigating the effects of low-dose exposure to harmful substances [7]. These approaches play a significant role in identifying sensitive endpoints of toxic effects and analyzing the mechanisms of toxicity [8]. Bioinformatic analysis involves computational methods for examining complex genetic profiles, which are particularly suitable for understanding cellular development, pre-clinical changes, and other biological and trivial toxicological changes [9]. Therefore, it is particularly suitable for investigating low-toxicity substances like GenX. In fact, as a novel and advantageous approach in biomedical research, an increasing number of toxicological studies are employing bioinformatic analysis to explore toxicology, particularly in terms of the toxicological mechanisms and sensitive toxicological endpoints. The authors’ previous study has identified the key genes for heterocyclic amine exposure by using multiple bioinformatic methods [10]. Previous studies on PFASs (per- and polyfluoroalkyl substances) and GenX have also employed bioinformatic tools [11,12]. These results indicate that bioinformatic analysis has become an important tool in the study of toxicity for PFASs, including GenX. However, these studies are mostly focused on a specific animal model or human cell samples, and the results may be limited to that species or batch of experiments, with limited extrapolation. There is still a need for new methods to integrate experimental genomic results across different species, thereby extrapolating to humans.

Transcriptome data analysis is a crucial step in understanding the genetic basis of various biological processes. When dealing with the batch effects, several algorithms have been developed such as the SVA (Surrogate Variable Analysis) algorithm and rank-in algorithm [13]. Rank-in has gained popularity in recent years because it is particularly useful for cross-platform comparisons of transcriptome data, as it ranks genes based on their expression levels across different platforms and then identifies common patterns of gene expression [14]. Here, we hypothesize that the rank-in algorithm may help in exploring the transcriptional profiles of different species exposed to GenX, as this will enable researchers to identify conserved genetic mechanisms and understand how these mechanisms contribute to species-specific traits [15].

In this study, we endeavored to investigate the transcriptional responses of biological samples from different species exposed to GenX, aiming to uncover key genes that are conserved across species. By collecting publicly available transcriptome data and employing the rank-in algorithm to integrate these datasets, we utilized machine learning approaches to identify seven pivotal genes that were subsequently validated across various species. Then, we applied the weighted gene co-expression network analysis (WGCNA) algorithm to explore the functional roles of these key genes and the toxicological effects of GenX exposure [16]. The findings from our research provide insights into the underlying genetic mechanisms and toxicological impacts of GenX exposure across different species. Furthermore, our results offer valuable references for future studies utilizing animal models to examine the toxicological effects of human exposure to GenX.

## 2. Materials and Methods

### 2.1. Transcriptome Data Collection

GEO (Gene Expression Omnibus, https://www.ncbi.nlm.nih.gov/geo/, accessed on 13 June 2024) is a public database providing functional genomic information from high-throughput gene expression data. We searched the GEO database to find the transcriptome data of human samples exposure to GenX with the following search terms ‘(GenX) AND “Homo sapiens”[porgn:__txid9606]’, or ‘(HFPO-DA) AND “Homo sapiens”[porgn:__txid9606]’. Then, the datasets were manually checked for final inclusion. Related datasets in mouse, rat, or zebrafish samples were also searched with similar terms and included. The GEO dataset was downloaded and imported into R (https://www.r-project.org/, accessed on 13 June 2024, version 4.4.0) by using the R package “GEOquery” [17] for further analysis.

### 2.2. The Merging of the Transcriptome Data with the Rank-In Algorithm

To merge the genes from different species, the genes from different datasets were firstly normalized with the trimmed mean of M values (TMM), a method used in transcriptome sequencing data analysis to normalize gene expression data across samples, by the R package edgeR [18]. Meanwhile, the genes were screened to select the gene homologs across species with the R package homologene. Then, the gene expression matrices containing the homologous genes across different datasets from different species were identified. The transcriptome datasets were merged by the rank-in algorithm following the instructions described by the authors of the rank-in algorithm [14], with the locally executable program supported by Python 3.9 downloaded at http://www.badd-cao.net/rank-in/index.html (accessed on 13 June 2024).

### 2.3. The Machine Learning Classification Algorithm

To investigate the key genes for GenX exposure, all the samples were divided into the GenX/nonGenX groups for the subsequent machine learning classification. To determine the key genes for classification algorithms, recursive feature elimination (RFE) was performed on the corresponding genes in the rank-in merged data by using the caret package in R. Then, the variables with the highest accuracy were selected for the subsequent classification algorithms. These key genes were validated with two classification algorithms, that is, random forest and support vector machine (SVM), with a 70/30 split for the training data and test data, in both the merged dataset and the datasets in different species. The random forest model was created and tested with the R package caret, and the SVM model was created and tested with the R package e1071.

### 2.4. Bioinformatic Analysis of the Mechanism of GenX Exposure and the Key Genes

To explore the gene that are correlated with GenX exposure and the key genes, weighted gene co-expression network analysis (WGCNA) was performed on the homolog genes in human dataset samples by using the R package WGCNA [15,16] as we previously described with minor revisions [10]. Considering that WGCNA quantifies the module–trait association by calculating the Pearson correlation and the trait variable should be numeric, the GenX/nonGenX traits were assigned a value 1 or 0 based on whether the sample was exposed to GenX or not. The other traits, such as the GenX exposure doses and time, were also included for calculation. The verbose value of all relative functions was set at 3 (the default value). Then, the genes in the key modules were annotated for Gene Ontology (GO), Kyoto Encyclopedia of Genes and Genomes (KEGG) and Disease Ontology with the R package culsterProfiler [19] and DOSE [20], as described in our previous work [10]. After that, the key genes identified by the machine learning algorithm and the top 50 genes, based on module membership in the WGCNA modules, were put in STRING (https://cn.string-db.org/, accessed on 13 June 2024) to construct the protein–protein interaction network of candidate genes [21,22]. Finally, analysis of the immune infiltration of the gene dataset was performed with the CIBERSORT approach [23] to reflect the potential alterations in immune cells induced by GenX.

### 2.5. Statistical Analysis and Plotting

A *p*-value < 0.05 was generally considered statistically significant unless otherwise stated. The correlation between variables were calculated with the R package Hmisc and the Pearson correlation method, and the correlation heatmaps were plotted with the R package pheatmap. The density plot of gene expression before and after rank-in was plotted by the limma package in R.

## 3. Results

### 3.1. Transcriptome Datasets

**Figure 1:**
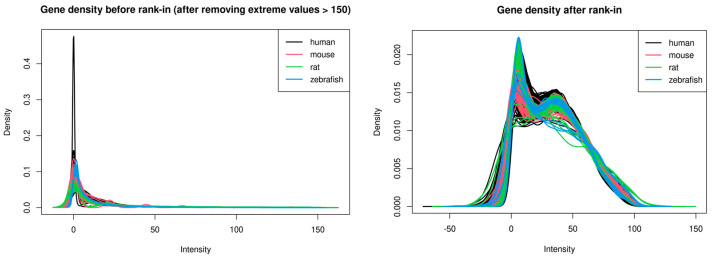
The gene density plot of each dataset before and after the rank-in. For density plot before rank-in, the gene expression values higher than 150 were removed. The plot without removing extreme values is shown in Supplementary Figure S1.

The search term ‘(GenX) AND “Homo sapiens”[porgn:__txid9606]’ on GEO returned three dataset results. One was excluded due to too few samples in which each dose had less than three replicates, and one was excluded because it was a mixture of human and mouse samples, and only the mouse samples were exposed to GenX. The final one was checked carefully, and the samples were not exposed to GenX alone but to a mixture of multiple PFASs. The search term ‘(HFPO-DA) AND “Homo sapiens”[porgn:__txid9606]’ on GEO returned one dataset result, GSE248251, which included 220 samples of human hepatocyte samples. Surprisingly, the dataset also contains 880 other samples from rat and mouse hepatocytes; among these samples, 220 wildtype B6129SF2/J mouse hepatocyte samples and 220 rat hepatocyte samples were also included. Given that GenX, like other PFASs, is present in aquatic environments and that zebrafish are utilized as a model organism in toxicological studies of GenX [24], we then searched the GEO dataset for GenX experiments performed on the zebrafish model, and one GEO dataset, GSE198976, with 19 samples was included. Samples from GSE248251 were exposed to GenX at different doses for different exposure times; samples from zebrafish were exposed to GenX at different doses for 72 h. The summary of these datasets is shown in Table 1, and the gene density plots of these datasets before and after the rank-in were shown in Figure 1.

We then obtained the homolog genes among the datasets of human, mouse, rat, and zebrafish. By performing homolog gene transformation in R and obtaining the intersected probes, 4838 genes were identified as common genes among the datasets of human, mouse, rat, and zebrafish, which are listed in Supplementary Table S1. Then, each dataset was normalized with TMM, and the 4838 genes were selected and combined with the rank-in algorithm to obtain the combined dataset for further analysis.

### 3.2. Machine Learning Identified Seven Key Genes for Distinguishing the GenX and Non-GenX Groups

The result of RFE on the merged dataset (Table 2), with samples classified as GenX and non-GenX groups, showed that seven variables reached the highest accuracy with the least number of variables. Therefore these seven genes, *TTR*, *ATP6V1B1*, *EPHX1*, *ITIH3*, *ATXN10*, *UBXN1*, and *HPX*, were considered key genes for distinguishing the GenX exposure group.

**Figure 2:**
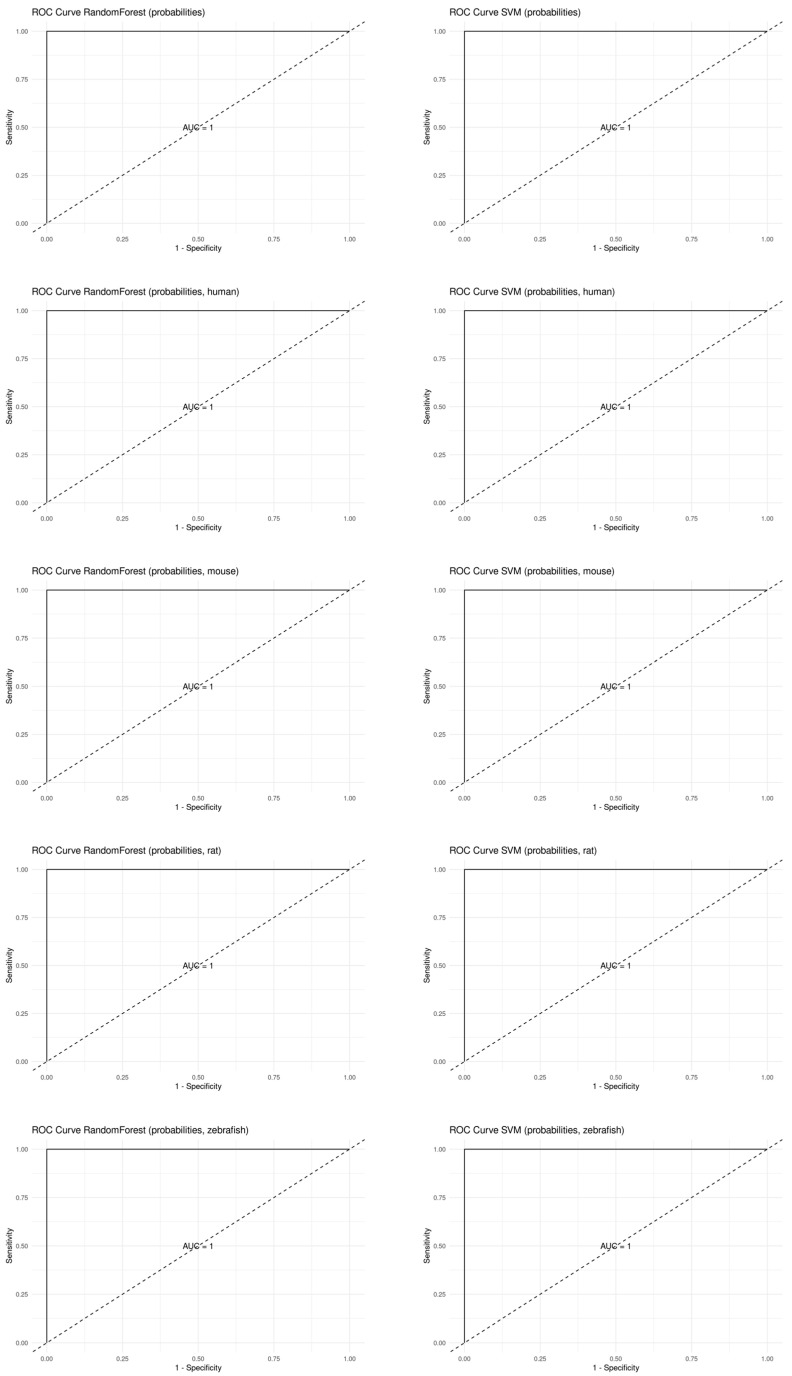
The receiver operating characteristic (ROC) curves of the machine learning algorithms with the ranked-in data.

To further verify whether the seven key genes can distinguish the GenX and non-GenX groups, we performed two classification algorithms: random forest and SVM, with ranked-in data in both all samples and samples of different species. The result showed that the seven genes reached a high accuracy at 1, in all samples and in samples of different species, as shown in Table 3 and Figure 2.

**Figure 3:**
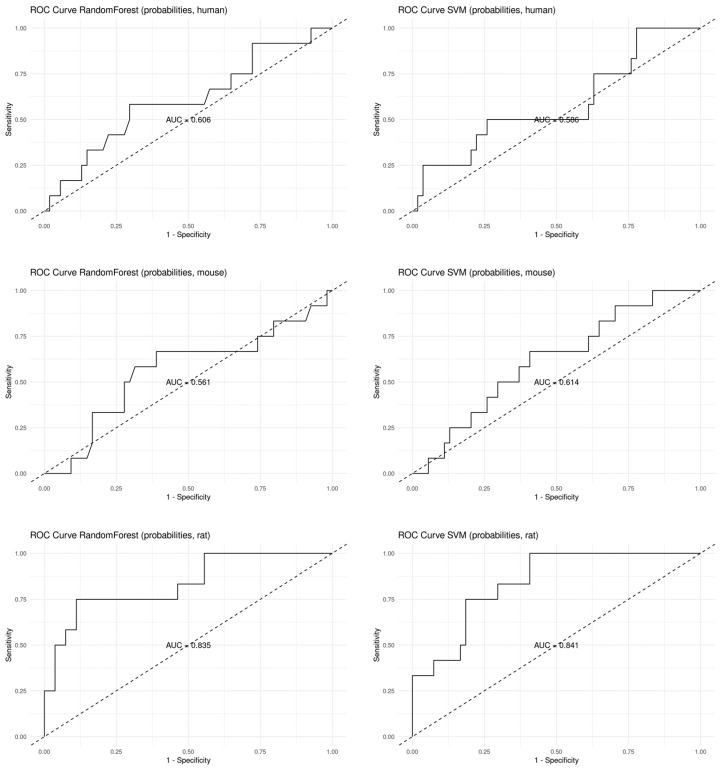
The receiver operating characteristic (ROC) curves of the machine learning algorithms on the data before rank-in. Curves in zebrafish were not plotted due to the low accuracy.

To further verify whether the seven genes were significant for distinguishing the GenX and non-GenX groups, the random forest and SVM classification were also performed on the datasets before rank-in. The results showed that the classification with the seven genes also achieved high accuracy in the human, mouse, and rat datasets before rank-in, ranging from 0.7879 to 0.8788, as shown in Table 4 and Figure 3. Taken together, these results suggest that the seven genes were the key genes for distinguishing the GenX and non-GenX groups, at least in the GSE248251 datasets of human, mouse, and rat.

### 3.3. The Gene Function and Network Analysis Revealed Mitochondrial Function and Metabolic Process as Being Potential Modulated by GenX and the Key Genes

**Figure 4:**
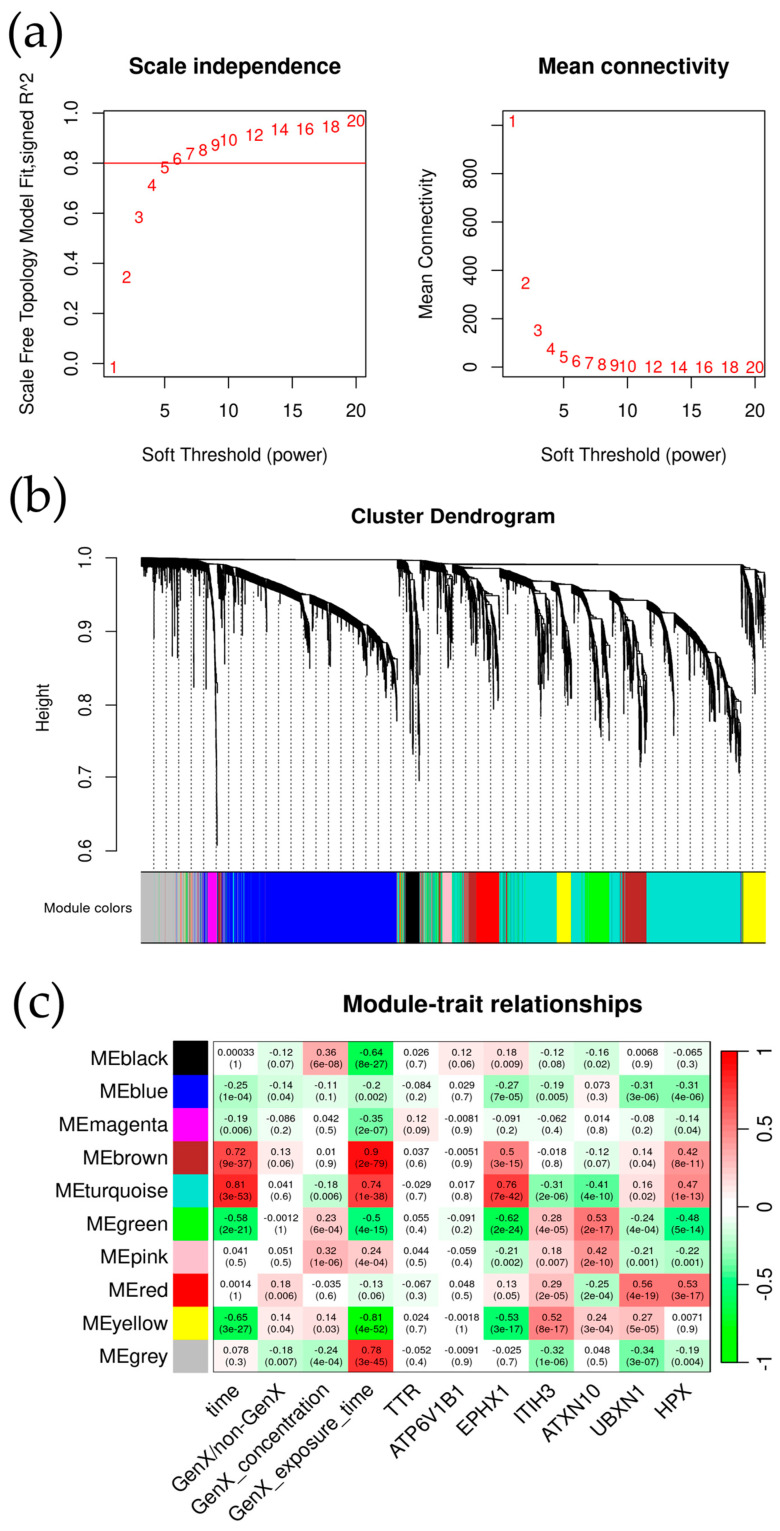
The result of WGCNA. (**a**) The result of soft-power selection. (**b**) The cluster dendrogram at the selected soft-power. (**c**) The module–trait relationships of the different modules and GenX exposure dose and time patterns and the seven key genes; the color in each cell indicates the correlation *r* value, ranging from −1 to 1 (color green to color red). Values in each cell were the *r* values, and the corresponding *p* values (In the bracket).

To further explore whether and how the seven key genes may be involved in the toxicity of GenX, we performed gene correlation network analysis and function annotation for the potential genes. Given that human exposure to GenX is what we focused on most, we used the human cell line data in GSE248251 with the 4838 common genes selected for further research. We initially performed WGCNA to divide the genes into different modules. After the soft power calculation, we set the soft-thresholding power at six so that the scale-free R-square reached >0.80 (Figure 4a,b). Then the 4838 genes were divided into 10 modules marked with different colors, as shown in Figure 4c. The result showed that the modules correlated with these key genes, especially five of the key genes *(EPHX1*, *ITIH3*, *ATXN10*, *UBXN1*, and *HPX*), were also correlated with GenX doses and exposure times, suggesting the dose- and time-response potentials of these genes. Among the gene modules, turquoise, green, and yellow were the modules correlated with GenX doses and exposure times that were also correlated with the five key genes (Figure 4c); the red module was the module that was significantly associated with GenX or non-GenX classification and was the module where *UBXN1* and *HPX* were (Figure 4c, Supplementary Table S2). Therefore, the turquoise, green, yellow, and red modules were enrolled in the subsequent study.

**Figure 5:**
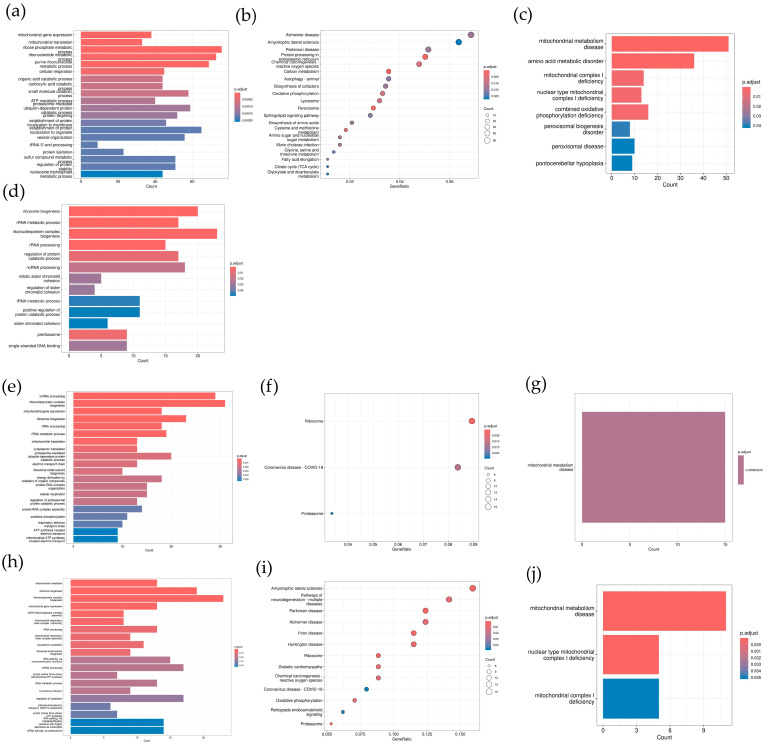
The gene annotation result of different modules. (**a**–**c**) The GO, KEGG, and Disease Ontology annotation of genes from the turquoise module; (**d**) the GO annotation of genes from the green module (KEGG and Disease Ontology returned no significant terms); (**e**–**g**) the GO, KEGG, and Disease Ontology annotation of genes from the yellow module; (**h**–**j**) the GO, KEGG, and Disease Ontology annotation of genes from the red module.

**Figure 6:**
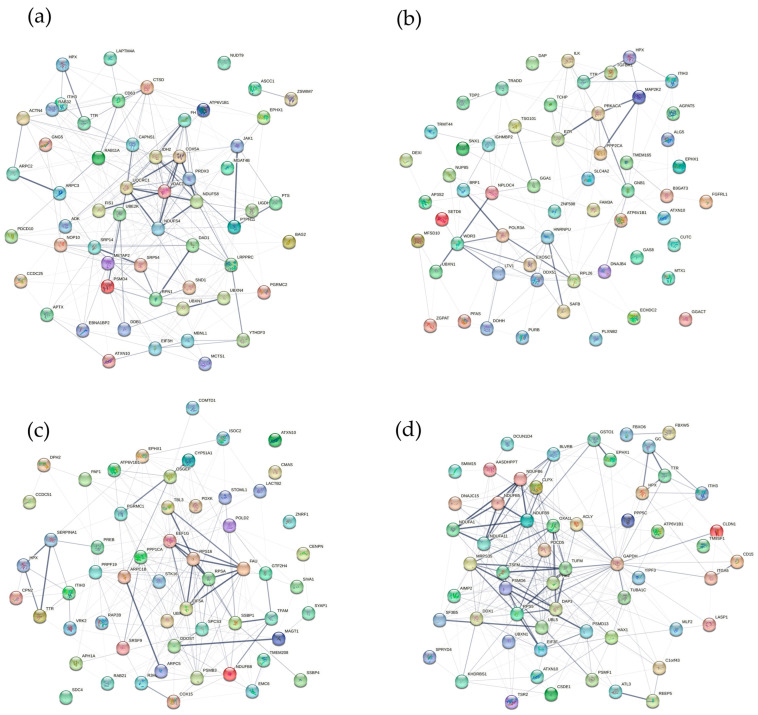
The network of proteins for key genes in the gene modules. The line thickness between network edges indicates the confidence, with low confidence at 0.15. (**a**) The top 50 module membership genes in the turquoise module and the 7 genes; (**b**) the top 50 module membership genes in the green module and the 7 genes; (**c**) the top 50 module membership genes in the yellow module and the 7 genes; (**d**) the top 50 module membership genes in the red module and the 7 genes.

The gene functions of genes from the turquoise, green, yellow, and red modules were annotated. As shown in Figure 5, all the turquoise, yellow, and red genes were annotated in mitochondrial and metabolic terms, such as mitochondrial translation, ATP metabolism, and carboxylic and amino acid metabolism, while the genes in the green module were annotated in RNA process and protein metabolism. These results suggest that GenX and the key genes, especially the five genes *EPHX1*, *ITIH3*, *ATXN10*, *UBXN1*, and *HPX*, were correlated with genes potentially modulating mitochondrial function and metabolic process. The gene network correlation between genes in the modules and the seven key genes is shown in Figure 6.

### 3.4. The Immune Function Showed No Dose-Response Relationship with GenX Exposure Doses

**Figure 7:**
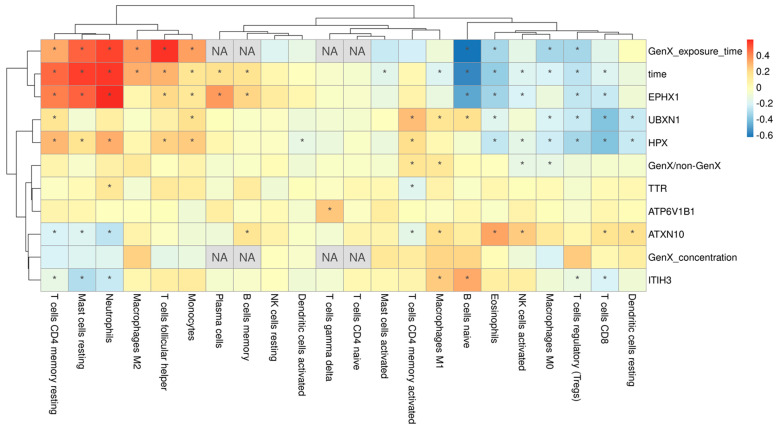
The immune infiltration result and the correlations with GenX exposure and key genes. “*” indicates a Pearson correlation with *p* value < 0.05. “NA” in cells indicates that the correlation is not applicable or too trivial between the immune parameter and the exposure/gene.

We finally performed immune infiltration analysis with the CIBERSORT approach to evaluate the potential of GenX to disrupt immune function. As shown in Figure 7, the immune infiltration result showed correlations with time and several key genes, but most of the parameters were not significantly correlated with GenX concentration, suggesting that immune function may not be dose-responsively correlated with GenX, which indicates that the immune system may not be a sensitive target of the toxicity of GenX.

## 4. Discussion

In the present study, we investigated the genetic mechanism underlying the GenX exposure by searching and analyzing the transcriptomes in the GEO database. By using the rank-in algorithm for merging different gene expression datasets and machine learning classification for samples exposed to GenX or not, we successfully identified seven key genes as the features for distinguishing the GenX-exposed group and non-GenX-exposed group, in both the rank-in combined dataset or the separate datasets of human, mouse, and rat, before and after the rank-in. The results suggested that the seven key genes *TTR*, *ATP6V1B1*, *EPHX1*, *ITIH3*, *ATXN10*, *UBXN1*, and *HPX* may play critical roles in the GenX exposure and toxic effects across human and animal models. Our research sheds light on the fundamental genetic pathways and toxicological consequences of GenX exposure across various species and provides crucial references for future investigations using animal models to assess the toxic effects of human exposure to GenX.

Transcriptome data analysis is an indispensable step towards deciphering the genetic underpinnings of various biological phenomena. Nonetheless, researchers frequently encounter the confounding issue of batch effects in transcriptome data. To address this challenge, a multitude of algorithms, including SVA and rank-in, have been devised to correct batch effects [13,14]. The rank-in algorithm, in particular, has proven invaluable for cross-platform comparisons of transcriptome data [15]. This algorithm operates by ranking genes based on their expression levels across diverse platforms, subsequently pinpointing consistent patterns of gene expression [14]. Therefore, the rank-in algorithm facilitates the comparative analysis of transcriptome data derived from different platforms, enabling the discovery of conserved biological pathways or regulatory networks. In our present study, we for the first time employed the rank-in algorithm to amalgamate transcriptome data from disparate species. The outcome demonstrated a commendable identification of key genes, a finding that was corroborated by applying machine learning methods to both the combined and individual datasets. Our findings suggest that the rank-in algorithm, when integrated with machine learning techniques, may serve as an effective strategy for elucidating the genetic mechanisms underlying transcriptome data across various species (Figure 2 and Figure 3). This approach is particularly pertinent to toxicological research, where animal models are frequently employed for evaluating toxicological effects.

After the machine learning process identified seven genes based on the GenX and non-GenX classification, we performed a correlation analysis of GenX exposure doses and times with the seven key genes and other genes. The correlation with gene modules distinguished by WGCNA showed that the turquoise, green, yellow, and red modules were closely correlated with GenX exposure and the key genes, especially the five genes *EPHX1*, *ITIH3*, *ATXN10*, *UBXN1*, and *HPX* (Figure 4). The correlation exhibited a high degree of coherence with the machine learning results, confirming the efficacy of the rank-in integrated machine learning approach in identifying key genes involved in GenX exposure. Among the five genes, *EPHX1* (Epoxide Hydrolase 1) encodes a member of the epoxide hydrolase family of enzymes, which are involved in the metabolism of many compounds, including drugs and xenobiotics. This gene plays a crucial role in the biotransformation and detoxification of potentially harmful compounds [25]. *ITIH3* (Inter-Alpha-Trypsin Inhibitor Heavy Chain 3) is part of the inter-alpha-trypsin inhibitor family, a group of proteins that circulate in the blood and are involved in the stabilization of the extracellular matrix [26]. They also have roles in wound repair and inflammation [27]. *ATXN10* (Ataxin-10) encodes a protein that belongs to the family of ataxin proteins, which are implicated in neurodegenerative disorders such as spinocerebellar ataxia and may have roles in transcriptional regulation and RNA processing [28]. *UBXN1* (UBX domain-containing protein 1) encodes a protein that contains a UBX domain, which is a motif found in proteins associated with the ubiquitin-proteasome system, and is responsible for degrading unneeded or damaged proteins in cells [29]. *HPX* (Hemopexin) is a plasma glycoprotein that is involved in the transport of heme to liver cells, where it is degraded and recycled [30]. Hemopexin helps to prevent heme-induced oxidative damage and is important in iron metabolism and regulation [31]. All five genes were not previously reported to be correlated with GenX. We further analyzed the function of key modules—turquoise, green, yellow, and red—and found that these four modules were mainly related to mitochondria and metabolic activities (Figure 5), while EPH1 has been reported to be correlated with PPARα and mitochondrial fatty acid degradation and glycolysis in antitumor research in a rat model [32]. Interestingly, *PPAR*α, as was focused on by the original authors of GSE248251, has been found to be a crucial toxic mechanism of GenX [33]. Although the calculation methods were different, our finding also support the conclusion of the original research of the authors of dataset GSE248251.

The issue of whether mitochondria serve as a toxic target for GenX has been shrouded in controversy. The United States Environmental Protection Agency (USEPA) has suggested that mitochondrial metabolism could be one of the targets affected by GenX exposure [34]. However, subsequent literature reviews have generally not found sufficient evidence to support claims of mitochondrial dysfunction associated with GenX [35]. Despite this, other research contends that there is indeed a connection between GenX exposure and mitochondrial disruption, indicating that further investigation is necessary to conclusively determine the relationship between GenX and mitochondrial health [36]. In the current study, we found by gene annotation that mitochondrial and metabolic activities were correlated with GenX exposure (Figure 5). Although our study does not provide conclusive evidence, our gene annotation analysis and network graphs (Figure 6) can offer research clues on the relationship between GenX toxicity and mitochondria, providing research value to resolve this controversial issue.

Finally, we explored the immune response to GenX with the CIBERSORT algorithm. CIBERSORT was developed for deconvoluting bulk gene expression data to estimate the composition and abundance of cell types in a mixed cell population [23]. I CIBERSORT was initially developed for characterizing the immune cell composition in tumor tissues. However, its application has since expanded to non-tumor tissues as well and now has become a valuable tool in immunology research [37]. In the present study, we utilized CIBERSORT to investigate the immune response to GenX in a human dataset. The results revealed that there were no significant immune changes associated with varying doses of GenX (Figure 7). Additionally, we applied CIBERSORT to the zebrafish dataset GSE198976, which similarly indicated that there were no immune changes correlated with GenX doses. However, recent studies in lung cells and oysters reported that several immune-related genes were regulated by GenX [38,39]. In fact, the key gene UBXN1 identified in the current research is also an immune-related gene that was reported to be involved in antiviral immune response [40]. In brief, whether GenX may exert immune-related toxicity and how to evaluate the effect of GenX on the immune system need further research.

There are limitations of the current study. One is that the transcriptome samples in zebrafish are not enough to support the machine learning calculation (Table 3 and Table 4). Second, in the WGCNA, most gene modules correlated with time and exposure time, which may because of the natural alterations in gene expression with the cell culture time. In future research, it may be necessary to develop improved computational methods to eliminate the changes in gene expression caused by time effects themselves, thereby more accurately identifying the key genes associated with chemical exposure.

## 5. Conclusions

In conclusion, our study identified seven genes—*TTR*, *ATP6V1B1*, *EPHX1*, *ITIH3*, *ATXN10*, *UBXN1*, and *HPX*—as key markers for distinguishing GenX exposure groups. These findings were derived from transcriptome datasets across different species using rank-based integrated machine learning algorithms. The underlying mechanisms of GenX toxicity appear to be linked to mitochondrial and metabolic activities, which may be modulated by these key genes. Future experimental research is necessary to further validate and explore the mechanisms of GenX toxicity.

## Figures and Tables

**Table 1:** The summary of the datasets included in the study.

GEO Dataset	Species	Number of Samples in Total	Number of Samples Exposed to GenX	Features of Exposure Patterns	Gene Expression Unit
GSE248251	human	220	40	dose; exposure time	count
GSE248251	mouse	220	40	dose; exposure time	count
GSE248251	rat	220	40	dose; exposure time	count
GSE198976	zebrafish	19	16	dose	TMM

**Table 2:** The result of RFE on the merged dataset with GenX and non-GenX classification.

Number of Variables	Accuracy	Kappa	Accuracy SD	Kappa SD
1	0.979122	0.936034	0.021961	0.065543
2	0.993706	0.97971	0.010136	0.032714
3	0.995833	0.986885	0.008784	0.027648
4	0.995789	0.986267	0.008878	0.028988
5	0.997917	0.993443	0.006588	0.020736
6	0.997917	0.993443	0.006588	0.020736
7	1	1	0	0
8	1	1	0	0
9	1	1	0	0
10	1	1	0	0
4838	1	1	0	0

**Table 3:** The classification accuracy of the random forest and SVM of the ranked-in data. The results are expressed as accuracy (95% confidence interval).

Algorithm	Integrated Dataset	Human	Mouse	Rat	Zebrafish
Accuracy in random forest model	1 (0.9819, 1)	1 (0.9456, 1)	1 (0.9456, 1)	1 (0.9456, 1)	1 (0.3976, 1)
Accuracy in SVM model	1 (0.9819, 1)	1 (0.9456, 1)	1 (0.9456, 1)	1 (0.9456, 1)	1 (0.3976, 1)

**Table 4:** The classification accuracy of the random forest and SVM on the data before rank-in. The results are expressed as accuracy (95% confidence interval).

Algorithm	Human	Mouse	Rat	Zebrafish
Accuracy in random forest model	0.803 (0.6868, 0.8907)	0.7879 (0.6698, 0.8789)	0.8788 (0.7751, 0.9462)	0.5 (0.0676, 0.9324)
Accuracy in SVM model	0.8182 (0.7039, 0.9024)	0.8182 (0.7039, 0.9024)	0.8182 (0.7039, 0.9024)	0.5 (0.0676, 0.9324)

## Data Availability

The data presented in this study are openly available in the Gene Expression Omnibus at https://www.ncbi.nlm.nih.gov/geo/ (accessed on 13 June 2024), reference number GSE248251; GSE198976.

## Supplementary Materials

The following supporting information can be downloaded at: https://www.mdpi.com/article/10.3390/toxics12070516/s1, Figure S1: the gene density plot before rank-in without removing extreme values; Table S1: the 4838 common genes list; Table S2: the module membership of the 7 key genes; Table S3: the performance measures of the classification models.
