# Supplementary figures and images for: Rank-In Integrated Machine Learning and Bioinformatic Analysis Identified the Key Genes in HFPO-DA (GenX) Exposure to Human, Mouse, and Rat Organisms
Source: Toxics. 2024 Jul 18;12(7):516. doi: 10.3390/toxics12070516 (PMC11280914; doi:10.3390/toxics12070516)

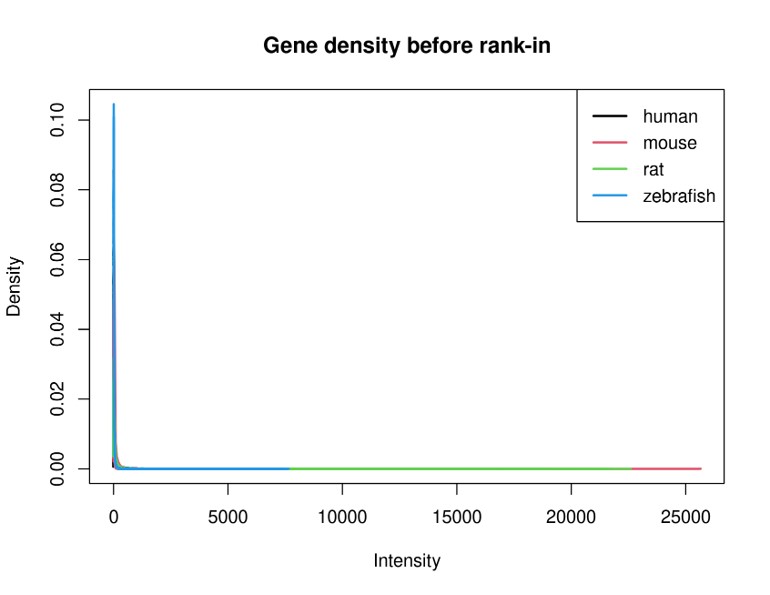

Supplement: Supplementary file 1 [file toxics-12-00516-s001.zip › Figure S1 the gene density plot before rank-in without removing extreme values.jpg]
